# Radiographic Features of the Maxillofacial Anomalies in Beta-Thalassemia Major: With New View

**DOI:** 10.29252/wjps.10.3.78

**Published:** 2021-09

**Authors:** Soheyla Bayati, Bijan Keikhaei, Mohammad Bahadoram, Mohammad-Reza Mahmoudian-Sani, Mohammad Vaneshani, Fatemeh Behbahani

**Affiliations:** 1 Department of Oral & Maxillofacial Radiology, Dental Faculty, Ahvaz Jundishapur University of Medical Sciences, Ahvaz. Iran; 2 Thalassemia & Hemoglobinopathy research center, Ahvaz Jundishapur University of Medical Science, Ahvaz, Iran

**Keywords:** Maxillo, Facial anomalies, Beta thalassemia, Blood transfusion

## Abstract

**BACKGROUND:**

Beta- thalassemia major causes the basic skeletal changes due to ineffective erythropoiesis in suffering patients. The aim of the study was to determine the frequency of maxillo-facial anomalies and the hemoglobin and ferritin levels in patients with beta-thalassemia major compared to the healthy control group.

**METHODS:**

The present study was performed on 72 beta- thalassemia major patients and 70 healthy control group in Ahvaz, Southwest Iran, from Jan 2014 to Mar 2015. Panoramic radiographs were taken using a standard procedure. The frequency of abnormalities including enlargement of bone marrow spaces, small maxillary sinuses, thickness of inferior mandibular cortex, prominent antegonial notch, absence of inferior alveolar canal and thin lamina dura, were determined by two Oral and Maxillofacial Radiologist. We also paid to identification of the relationship between abnormalities frequency and hemoglobin and ferritin levels during previous 6 months in thalassemia patients.

**RESULTS:**

The mean age of case and control groups was 18.6±7.25 and 17 ± 6. 55 yr, respectively. The frequency of abnormalities in the case and control groups was as follows, enlargement of bone marrow spaces [69 (95.8%) vs 3 (4.3%)], small maxillary sinuses [45 (62.5%) vs 1(1.4%)], reduced thickness of inferior mandibular cortex [21(29.2%) vs 6 (8.6%)], prominent antegonial notch [10 (13.9%) vs 2 (2.9%)], absence of inferior alveolar canal [68(94.4%) vs 41(58.6%)] and thin lamina dura [40 (55.6%) vs 5 (7.1%)].

**CONCLUSION:**

The all above mentioned abnormalities in patients with beta-thalassemia major was higher than the control group. Moreover, the frequency of maxillo-facial abnormalities decreased by increasing hemoglobin and decreasing ferritin.

## INTRODUCTION

Thalassemia is the most common known single gene disorder in the wide world, which has a variety of types^1^. Beta-thalassemia major is the most severe form of beta thalassemia which can be life-threatening, if remains untreated. Iran and other Middle East countries are the endemic areas for this disease. In Iran there are 25,000 patients with beta-thalassemia major^1^^, ^^2^. 

The etiology of this disease is a defect in hemoglobin beta protein synthesis that leads to reduced hemoglobin, increased ferritin level, extensive hemolysis, and iron overload^3^^, ^^4^. Therefore, hyperplasia and maxillofacial expansion develops that can lead to maxillofacial deformities^5^^, ^^6^. The impact of thalassemia on the jaw and skull radiograph include decreased of general bone density due to bone marrow overgrowth, thin cortical bone, absence of inferior alveolar canal, and small maxillary sinuses^7^^, ^^8^. 

Blood transfusion as a key treatment is applied to prevent of the skeletal changes in patients. Hemoglobin concentration must be higher than 10 for arrived to this aim^9^. Unfortunately, despite numerous clinical studies on patients with changes in the maxillofacial area, a few investigations had studied maxillo-facial radiological abnormalities. On the other hand, to the best of our knowledge, our study had the largest sample size comparison with other studies. It also appears to be no study has been paid directly to the relationship between hemoglobin and ferritin concentrations with the maxillo-facial abnormalities. 

We aimed to compare the frequency of maxillo-facial abnormalities and serum levels of hemoglobin and ferritin in patients with beta-thalassemia major with the control group. 

## MATERIAL AND METHODS

*Ethics Approval*

This trial was approved by the Ahvaz Jundishapur University of Medical Sciences Ethics Committee (approval no: IR.ajums.REC.1393.276). Informed consent was taken from all participants in accordance with the provisions of the Declaration of Helsinki, after approval of the Ethics Committee of Ahvaz Jundishapur University of Medical Sciences. 

This case-control study was performed in Ahvaz, Southwest Iran, from Jan 2014 to Mar 2015. The case group included 72 patients of definitive diagnosis of beta-thalassemia major under transfusion therapy on a monthly basis (according to the treatment protocol). The control group also was selected from individuals referred to the Oral Medicine and Radiology Department of Ahvaz Jundishapur University of Medical Sciences. Inclusion criteria were no history of blood diseases or syndromes. Demographic data and the mean serum hemoglobin and ferritin concentrations of patients during past 6 months were collected from the medical records. The panoramic radiographs were taken in both case and control group. They were prepared with cranex D (PM 2010, Finland, Sordex). The evaluation of radiographic images was performed on the monitor (SONY VAIO D13213CX) by two oral radiologists, separately. The radiographs were examined for any radiological abnormalities include enlargement of bone marrow spaces, small maxillary sinuses, thickness of inferior mandibular cortex, prominent antegonial notch, and absence of inferior alveolar canal and thin lamina dura. 

Types of abnormalities were classified by the definitions of Hazza et al.’s study10 except for thin lamina dura as White and Pharoah textbook definition11. In addition, we indicated the effects of gender, hemoglobin and ferritin concentrations on maxillo-facial radiologic abnormalities in the form of a probabilistic model proposed by regression logistic model, for the first time. Investigators (oral radiologists) reviewed together again radiographs in cases where there was disagreement among them. Finally, observers agreed on all graphics interpretations and reported the same interpretations. The significant level was considered as *P*<0.05. The Chi-Square test was used to compare the relative frequency of each parameter in the control and case groups. Data were analyzed using SPSS 18 (Chicago, IL, USA).

## RESULTS

Among 72 patients, 29 (40.3%) were female and 43 (59.7%) were male. Similarly, 70 patients were entered to the control group. There was a similar distribution of age and gender in both groups (Table 1). 

The patients were divided into 3 subgroups ages, including less 10, 10-20, and higher 20 yr of age. There was no statistically significant relationship between the age and frequency of maxillo-facial abnormalities in three age groups. Moreover, there was no significant difference between the maxillo-facial abnormalities and gender of patients. The most frequency of maxilla-facial deformities, in patients with beta- thalassemia major vs. control group are shown in Table 2. There was no significant correlation between the median hemoglobin and maxilla-facial abnormalities (Table 3). Similarly, there was no significant relationship between the mean serum ferritin and maxillofacial deformities, except thin lamina dura anomaly. We also used logistic regression model for prediction the occurrence of types of anomalies based on different factors. These factors included age, gender, hemoglobin, and ferritin concentrations (Table 4-9). The probability risk for the enlargement of bone marrow spaces in patients increased by 38% (OR=1.38) per each year increasing the age. The occurrence of reduced thickness of inferior mandibular cortex abnormality was 2.2 more frequent in males than females (OR=0.44). In addition to, thin lamina dura was 2.3 more frequent in females (OR=2.34). Moreover, for each 1-unit decrease in hemoglobin level, the chance of reduced thickness of inferior mandibular cortex, absence of inferior alveolar canal, and prominent antegonial notch increased 35% (OR=1.35), 49% (OR=1.49), and 85% (OR=1.85), respectively.

## DISCUSSION

The nature of beta-thalassemia major is in a manner that may cause major changes in morphology especially in maxillofacial organs as part of the skeletal system. Although, maxillofacial malformations are well known in patients with beta-thalassemia major, a few studies have been performed on the frequency of radiological abnormalities of patients^10^^, ^^12^. Unlike some studies on radiographic images of thalassemia, examiners decided after were agreed together in all the details of the results give the unit results of their observation. 

We decided to use this method since the investigated abnormalities in our study are more visible than other abnormalities and the reading of the investigated abnormalities is relatively easy. This method may be more efficient compared to existing and conventional methods that examiners have nothing relationship together. This is because after the observer’s arguments against the agreement after they have seen what will be done. In this study, the observers on all the details of the abnormalities are found to agree 100%, which in itself is new. Furthermore, few studies had examined the maxillofacial deformities in individuals older than 18 yr in patients with thalassemia major; and our study had the largest sample size comparison with other studies^12^. In addition, to the best of our knowledge, we studied the relation between frequency of these abnormalities with the median ferritin and hemoglobin level during the last 6 months ago, in the patients affected, for the first time. According to the present study, maxilla-facial malformations had the highest frequency, in the enlargement of bone marrow spaces, absence of inferior alveolar canal, small maxillary sinuses, thin lamina dura, reduced thickness of inferior mandibular cortex, prominent antegonial notch, respectively. With little difference, Hazza and Jamal introduced reduced thickness of inferior mandibular cortex, absence of inferior alveolar canal, small maxillary sinuses, thin lamina dura, enlargement of bone marrow spaces and prominent antegonial notch as the highest frequency, respectively^10^. 

In our study, thin lamina dura was seen in 40 (55.6%) patients with thalassemia and in 5(7.1%) patients of the control group. This result was concordance with other studies that reported deformity in 46% and 40% of patients with thalassemia, respectively^10^^, ^^12^. Small maxillary sinuses were reported in 45 thalassemic patients (62.5%) and in 1(1.4%) individual in the control group in our study. Similarly, in some studies were seen in 50% and 56% of patients with thalassemia, respectively^10^^, ^^12^. The prominent antegonial notch in our study was in 10 (13.9%) patients with thalassemia and in 2 (2.9%) samples of the control group. That in accordance with our results, in another study 9 (18%) of patients with thalassemia and 7 (14%) of the control group had abnormalities, respectively^10^. Although Hazza and Jamal introduced the reduced thickness of inferior mandibular cortex in all patients with thalassemia major, we had a completely contradict their findings. In our study, it was observed only in 21(29.2%) samples^10^. Probably part of the difference was for this reason that we considered the values less than 3 mm as the reduced thickness of inferior mandibular cortex in adults (9) in contrast to other studies^10^^,^^12^. 

Complete absence of inferior alveolar canal was seen in 33 (66%) patients with thalassemia and in 23 (46%) cases in other studies. Contrast to this finding, we demonstrated 68(94.4%) of cases had this abnormality. The difference in this study may be related to this fact that the higher incidence of this disease in our studied population. Because in healthy subjects of our study, 41 (58.6%) samples had these disorders. However, this deformity was seen another study only in 1(2%) of the control group^10^^, ^^12^. The enlargement of bone marrow spaces was introduced in 21 (42%) patients with thalassemia and others reported this number, 27 (54%). However, in our study, 69 (95.8%) patients, compared with 3 (4.3%) of the control group showed this abnormality^10^^, ^^12^. This can be contributed to higher age mean of individuals. Because the mean age of patients in our study was 18.57 ± 7.25 yr and significantly higher than in other studies (7.15 ± 0.9 ^10^, 14.34± 5.83 ^12^)’ this may explain the results^11^. However, in our study, there was not a significant correlation between age and any maxillo-facial abnormalities. This can be related to higher frequency of individuals in the age group 10-20 yr in subjects. 

The thalassemia causes wide systemic changes on skeletal system and the various body organs^13^^,^^14^. However, it does not seem like a study to examine the relationship between hemoglobin and ferritin and maxillofacial anomalies. The effect of blood transfusion regimen (based on amount of blood transfused) was evaluated on the head and teeth in children with severe thalassemia. According to this study, most patients with beta-thalassemia major who received a high blood transfusion regimen had lower anomalies rate than low blood transfusion regimen^15^.

**Table 1:** Age and sex distribution of the case and control group

Variable		Beta thalassemia major	Control	P-value
Gender	Female (%)	29(40.3)	34(48.6)	0.32
	Male (%)	43(59.7)	36(51.4)	
Age		18.57±7.25	16.97 ± 6.55	0.17

**Table 2:** The frequency and percent of radiographic deformities in patients with major beta thalassemia compared with the control group

Radiographic deformities	Beta- thalassemia major N(%)	Control N(%)	*P*-value
Enlargement of bone marrow spaces	69(95.8)	3(4.3)	<0.001
Absence of inferior alveolar canal	68(94.4)	41(58.6)	<0.001
Small maxillary sinuses	45(62.5)	1(1.4)	<0.001
Thin lamina dura	40(55.6)	5(7.1)	<0.001
Reduced thickness of inferior mandibular cortex	21(29.2)	6(8.6)	0.002
Prominent antegonial notch	10(13.9)	2(2.9)	0.03

**Table 3:** Relationship between the median of hemoglobin and ferritin serum and maxillofacial abnormalities in patients with major beta thalassemia

Type of Anomaly	Hb	P-Value	Ferritin	*P*-value
Enlargement of bone marrow spaces	7.85 (6.2-11.6)	0.22	3100 (149 -5950)	0.46
Thin lamina dura	7.85 (6.2-11.6)	0.42	3100 (200 -5950)	0.003
Small maxillary sinuses	7.85(6.20-11.30)	0.95	3110(375-5147)	0.96
Reduced thickness of inferior mandibular cortex	7.70(6.20-9.60)	0.31	3247(1000-5147)	0.48
Prominent antegonial notch	7.65 (6.80-8.70)	0.20	3041(1000-4900)	0.77
Absence of inferior alveolar canal	7.78 (6.20-11.60)	0.30	3100(149-5950)	0.47

**Table 4:** The logistic regression model for Enlargement of bone marrow spaces based on age, gender, Hb and ferritin

Enlargement of bone marrow spaces	β	Sig	Odds Ratio
Age	0.32	0.048	1.38
Hb	0.57	0.53	1.76
Ferritin	-0.001	0.18	0.99
gender	-0.89	0.55	0.41

**Table 5:** The logistic regression model for thin lamina dura based on age, gender, Hb and ferritin

Thin lamina dura	β	Si g	OR
Age	0.02	0.58	1.02
Hb	0.32	0.23	1.38
Ferritin	0.0	0.47	1
gender	0.85	0.10	2.34

**Table 6:** The logistic regression model for Small maxillary sinuses based on age, gender, Hb and ferritin

Small maxillary sinuses	β	sig	OR
Age	-0.042	0.26	0.959
Hb	0.005	0.98	1.005
Ferritin	0.0	0.83	1.00
gender	-0.38	0.46	0.685

**Table 7:** The logistic regression model for Reduced thickness of inferior mandibular cortex based on age, gender, Hb and ferritin

Reduced thickness of inferior mandibular cortex	β	sig	OR
Age	-0.027	0.49	0.973
Hb	-0.3	0.29	0.74
Ferritin	0.0	0.42	1
gender	-0.806	0.16	0.447

**Table 8:** The logistic regression model for Prominent antegonial notch based on age, gender, Hb and ferritin

Prominent antegonial notch	β	Sig	OR
Age	0.035	0.51	1.036
Hb	-0.62	0.16	0.54
Ferritin	0.0	0.97	1
gender	-0.546	0.44	0.58

**Table 9:** The logistic regression model for Absence of inferior alveolar canal based on age, gender, Hb and ferritin

Absence of inferior alveolar canal	β	sig	OR
Age	-0.074	0.38	0.93
Hb	-0.394	0.37	0.67
Ferritin	0.0	0.59	1
gender	-1.002	0.41	0.367

## CONCLUSION

In our study, there was no significant relationship among the frequency of maxillofacial anomalies and mean hemoglobin and ferritin. This may be related to lower sample size of individuals with low hemoglobin. There was a significant relationship between blood transfusion regimen and reduction of maxillofacial anomalies. Moreover, the frequency of maxillo-facial abnormalities decreased by increasing hemoglobin and decreasing ferritin.

## CONFLICT OF INTEREST

The authors declare that there is no conflict of interest.

## References

[1] De Sanctis V, Kattamis C, Canatan D (2017). β-Thalassemia Distribution in the Old World: an Ancient Disease Seen from a Historical Standpoint. Mediterr J Hematol Infect Dis.

[2] Khodaei GH, Farbod N, Zarif B, Nateghi S, Saeidi M (2013). Frequency of thalassemia in Iran and Khorasan Razavi. Int J Pediatr.

[3] Rechavi G, Rivella S (2008). Regulation of iron absorption in hemoglobinopathies. Curr Mol Med.

[4] Nienhuis AW, Nathan DG (2012). Pathophysiology and Clinical Manifestations of the β-Thalassemias. Cold Spring Harb Perspect Med.

[5] Helmi N, Bashir M, Shireen A, Ahmed IM (2017). Thalassemia review: features, dental considerations and management. Electron Physician.

[6] Amirabadi F, Saravani S, Miri-Aliabad G, Khorashadi-Zadeh M (2019). The Association between Dental Health Status and Oral Health-Related Quality of Life of Children Diagnosed with beta-Thalassemia Major in Zahedan City, Iran. Int J Pediatr.

[7] Hazza’a A, Al-Jamal G (2006). Radiographic features of the jaws and teeth in thalassaemia major. Dentomaxillofac Radiol.

[8] Valizadeh N, Farrokhi F, Alinejad V (2014). Bone density in transfusion dependent thalassemia patients in Urmia, Iran. Iran J Ped Hematol Oncol.

[9] Liumbruno G, Bennardello F, Lattanzio A, Piccoli P, Rossetti G (2009). Recommendations for the transfusion of red blood cells. Blood Transfus.

[10] Hazza’a AM, Al-Jamal G (2006). Dental development in subjects with thalassemia major. J Contemp Dent Pract.

[11] White SC, Pharoah MJ ( 2018). White and Pharoah’s Oral Radiology E-Book: Principles and Interpretation.

[12] Kashid AL, Kumbhare S, Sathawane R, Mody R (2013). Comparative evaluation of radiographic features of jaws and teeth on opg (orthopentamogram) in thalassemia major patients and normal individuals. Int J Curr Res.

[13] Cutando SA, Gil MJ, López-González GJD (2002). Thalassemias and their dental implications. Med Oral.

[14] Amini F, Jafari A, Eslamian L, Sharifzadeh S (2007). A cephalometric study on craniofacial morphology of Iranian children with beta‐thalassemia major. Orthod Craniofac Res.

[15] Jirarattanasopa V, Hooncharoen P, Mekaewkunchorn A, Torcharus K (2009). Effect of different transfusion regimens on craniofacial appearance and dentition in severe thalassemic children. Southeast Asian J Trop Med Public Health..

